# The Safety of Pre-Filter Heparin for Circuit Clotting Prevention in Continuous Renal Replacement Therapy: A Retrospective Evaluation

**DOI:** 10.3390/jcm15176502

**Published:** 2026-08-22

**Authors:** Thomas Diaz, Traci Grucz, Stephanie Davis, John Lindsley, Kathryn E. Dane, Hailey Burns, Samir Gautam, Gianna Iantosca

**Affiliations:** 1 Department of Pharmacy, The Johns Hopkins Hospital, Baltimore, MD 21287, USA; tediaz456@gmail.com (T.D.); tklotz1@jhmi.edu (T.G.); sdavis87@jhmi.edu (S.D.); jlindsl1@jhmi.edu (J.L.); kdane2@jhmi.edu (K.E.D.); hburns5@jh.edu (H.B.); giantos1@jhmi.edu (G.I.); 2 Department of Medicine, Division of Nephrology, Johns Hopkins University, Baltimore, MD 21287, USA

**Keywords:** bleeding, renal replacement therapy, heparin, pre-filter, filter lifespan, CRRT

## Abstract

**Background**: Circuit clotting is a common complication of continuous renal replacement therapy (CRRT). Pre-filter unfractionated heparin (UFH) is widely used to maintain circuit patency, but real-world data describing its safety and efficacy are limited. **Methods**: This retrospective, single-health-system study evaluated critically ill adults receiving pre-filter UFH for CRRT clotting prevention between 2021 and 2025. The primary outcome was clinically relevant major bleeding, adapted from International Society on Thrombosis and Haemostasis criteria. Secondary outcomes included pre-filter UFH dosing and monitoring patterns and CRRT filter outcomes. Categorical and continuous variables were summarized descriptively. Associations with bleeding were explored using Fisher’s exact tests. The times to bleeding and filter failure were evaluated using Kaplan–Meier analyses. **Results**: Of 96 screened patients, 77 met the inclusion criteria. Clinically relevant major bleeding occurred in 18 patients (23%), with no characteristics significantly associated with bleeding. The cumulative probabilities of bleeding at 24, 48, 72, and 96 h were 1.3%, 8.4%, 23.8%, and 35.3%, respectively. The median pre-filter UFH treatment duration was 72 h (IQR 37–137). Fifty-one patients (66%) experienced CRRT filter failure, with a median time to first filter failure of 36 h (95% CI 26.8–51.3). **Conclusions**: Clinically relevant major bleeding and filter failure were common during pre-filter UFH therapy in this cohort. Bleeding continued to occur with longer pre-filter UFH exposure, supporting close monitoring for bleeding during prolonged therapy. These findings provide real-world characterization of pre-filter UFH use but do not establish comparative safety. Larger prospective studies are needed to optimize pre-filter UFH dosing and monitoring.

## 1. Introduction

Continuous renal replacement therapy (CRRT) is frequently required in critically ill patients with acute kidney injury to correct metabolic derangements while maintaining hemodynamic stability [1]. However, CRRT is inherently limited by the risk of circuit filter clotting, leading to interruption and, thus, reduced solute clearance, impaired fluid balance, and increased healthcare costs [2]. As a result, anticoagulation strategies are critical to optimizing circuit patency.

Unfractionated heparin (UFH) is one of the most commonly used anticoagulants during CRRT due to its low cost and ease of administration [2,3]. As a non-dialyzable, short-acting agent that potentiates antithrombin, UFH has been shown to prolong CRRT circuit lifespan [3,4]. When used for circuit patency, UFH is typically administered as a continuous pre-filter infusion [3]. However, its use in critically ill patients is often complicated by substantial pharmacokinetic and pharmacodynamic variability. Unfractionated heparin exhibits non-specific binding to plasma proteins and cellular surfaces, and critical illness is associated with reduced antithrombin levels and increased acute-phase reactants, all of which contribute to unpredictable anticoagulant effects. Furthermore, UFH carries a risk of heparin-induced thrombocytopenia, further complicating its use [4]. These factors may result in either inadequate circuit anticoagulation or increased risk of bleeding. Reported rates of bleeding with pre-filter UFH vary widely from approximately 10 to 50% [5], demonstrating the variability in patient populations, dosing strategies, and outcome definitions.

Given these limitations of UFH, alternative anticoagulation strategies, such as regional citrate anticoagulation (RCA), have been increasingly adopted [2,3,4,6]. The Kidney Disease Improving Global Outcomes (KDIGO) acute kidney injury clinical practice guidelines recommend RCA as the preferred anticoagulant administered pre-filter for CRRT in patients without contraindications [3]. This recommendation is derived from multiple randomized controlled trials comparing citrate to heparins, demonstrating improved filter lifespan and reduced bleeding complications [7,8,9,10,11]. Among more recent evidence is that from the RICH trial, a large randomized controlled trial demonstrating improved filter lifespan and fewer bleeding complications with RCA [12], and the ORCA trial, a 10-year, large retrospective analysis demonstrating longer filter lifespans and lower transfusion requirements with RCA, though increased metabolic complications [13].

Despite these data, UFH remains widely used in clinical practice [3,14]. Barriers to implementing RCA-based protocols include the risk of metabolic derangements, need for specialized monitoring, and potential protocol complexity [15]. At many institutions, UFH remains the default pre-filter anticoagulation strategy for CRRT filter clotting prevention. Real-world data specifically evaluating outcomes related to a pre-filter UFH dosing strategy remain limited, as most studies have focused on comparisons between anticoagulation agents.

The primary objective of this study was to evaluate the incidence of clinically relevant major bleeding in critically ill adult patients receiving pre-filter UFH during CRRT. Secondary objectives included characterization of pre-filter UFH dosing and monitoring patterns and evaluation of circuit failure outcomes. By evaluating local pre-filter UFH prescribing patterns and associated safety outcomes, this study aims to inform safer anticoagulation strategies and optimize CRRT clotting management in critically ill patients.

## 2. Methods

This retrospective, multi-site, single-health-system cohort study consisted of adult patients receiving pre-filter UFH for CRRT clotting prevention between 27 April 2021 and 31 July 2025. Eligible patients were at least 18 years of age, were admitted to any ICU, had a dialysate bag order in combination with a prescriber-managed UFH order that contained pre-defined administration instructions for CRRT clotting management, and received pre-filter UFH for a minimum of 6 h. Patients were excluded if they received simultaneous therapeutic anticoagulation or received pre-filter UFH for less than 6 h. The end of therapy was defined as the time of pre-filter UFH order discontinuation, an interruption of more than 24 consecutive hours in pre-filter UFH as documented on the medication administration record, transition to an alternative RRT modality (i.e., prolonged intermittent renal replacement therapy), or fulfillment of any exclusion criteria. If a patient had multiple instances of pre-filter UFH administration during their index admission (i.e., pre-filter UFH was restarted after the end of therapy was documented), only the first instance was collected. Data were collected through manual chart review of patient electronic health records. The hospital’s Institutional Review Board acknowledged this study as a quality improvement project.

Continuous renal replacement therapy was delivered as continuous venovenous hemodialysis (CVVHD) using NxStage CAR-500 filters (NxStage Medical, Inc., Lawrence, MA, USA), with a typical blood flow rate of 200–300 mL/min. There was no institutional protocol specifying criteria for the initiation of pre-filter UFH. Initiation was based on the nephrologist or primary team’s clinical judgement and was generally reserved for patients with recurrent filter clotting or a perceived high risk of circuit clotting. Pre-filter UFH was typically initiated as a prescriber-managed, weight-based fixed starting dose of 6 units/kg/h administered via the CRRT pre-filter port, although dosing variations occurred at clinician discretion. The activated partial thromboplastin time (aPTT) was obtained daily for safety monitoring, but heparin titration to an aPTT goal is not routine. Additional aPTT measurements could be obtained based on clinical context.

Given that CRRT is an indication for an ICU level of care at our institution, the study population consisted exclusively of ICU patients. Patients without a predominant cardiovascular, surgical, burn-related, neurological, or oncological critical illness comprised the patients receiving care in the medical ICU. The collected variables included the pre-filter UFH duration and dose, aPTT abnormalities, age, ICU type, concomitant venous thromboembolism (VTE) prophylaxis or antiplatelet therapy, aspartate aminotransferase (AST) or alanine aminotransferase (ALT) elevations greater than five times the upper limit of normal during therapy, and platelet counts less than 50 × 10^3^/mm^3^ during therapy. Outcome-related variables included clinically relevant major bleeding and CRRT filter events.

The primary endpoint was the incidence of clinically relevant major bleeding: fatal bleeding, and/or symptomatic bleeding in a critical area or organ, and/or bleeding causing a fall in hemoglobin of at least 2 g/dL or leading to transfusion of at least 2 units of red blood cells within a 24 h period but not within 6 h of a procedure in the operating room. This definition was modified from the International Society on Thrombosis and Haemostasis (ISTH) major bleeding criteria [16]. Potential bleeding events identified during initial chart review were subsequently reviewed by three pharmacists who reached consensus regarding whether the bleeding criteria were met.

Secondary endpoints included pre-filter UFH dosing and monitoring patterns and CRRT filter outcomes. Alterations in aPTT were defined as a change in aPTT greater than two times baseline, a therapeutic aPTT within the range of 50 to 80.9 s, or a supratherapeutic aPTT greater than 80.9 s. Because aPTT measurements were obtained as part of routine clinical care, the collection times and number of measurements were not standardized across patients.

CRRT filter failure was determined based on documentation in the electronic health record or UFH medication administration record. Nursing flowsheet documentation of “rinseback unsuccessful” was included as a filter failure, unless there was an explicit nursing communication with an alternative explanation for the rinseback failure. Only the first filter event during the pre-filter UFH exposure period was included in the time-to-event analysis.

### Statistical Analysis

Categorical and continuous variables were summarized descriptively using percentages and measures of central tendency and spread. The primary bleeding endpoint was analyzed descriptively as the proportion of patients with a bleeding event. Patient and treatment characteristics were evaluated for unadjusted associations with clinically relevant major bleeding using two-sided Fisher’s exact tests as an exploratory analysis.

Time to first bleeding event was evaluated descriptively and using a Kaplan–Meier Survival Curve. Patients with a bleeding event were considered to have an event at the time of the first bleed, and patients without bleeding were censored at pre-filter UFH discontinuation, which represented the end of their eligible treatment period per chart review. The cumulative probabilities of bleeding were analyzed at various time points. A post hoc 48 h sensitivity analysis was performed for patients who remained on pre-filter UFH without a bleeding event at 48 h in order to characterize subsequent bleeding after the landmark.

Similarly, time to first CRRT filter failure was evaluated using a Kaplan–Meier Survival Curve. Patients with a filter failure were considered to have an event at the time of the first documented filter failure, and patients without filter failure were censored at pre-filter UFH discontinuation, which represented the end of their eligible treatment period per chart review. Since unsuccessful rinseback was collected but may not be specific for a true filter clot, a post hoc sensitivity analysis restricted the outcome to confirmed filter clotting. In this analysis, patients with an unsuccessful rinseback as their first filter event were censored at the time of the event because subsequent filter events were not systematically collected.

All statistical analyses were performed using Stata statistical software version 18.0 (StataCorp, College Station, TX, USA). *p*-values ≤ 0.05 were considered statistically significant.

## 3. Results

Of the 96 patients who received pre-filter UFH during the study period, 77 met the inclusion criteria. The most common reasons for exclusion were pre-filter UFH duration less than 6 h and non-CRRT dialysis modality. The median age was 61 years old (IQR 47–70), 66% were male patients, and the most common units to use pre-filter UFH were the cardiovascular surgical intensive care unit (CVSICU) and the medical intensive care unit (MICU). The median weight-adjusted starting dose was 535 units/h (IQR 462–706), and the median pre-filter UFH duration was 72 h (IQR: 37–137). Overall, 24 patients (31%) experienced at least one of the pre-specified aPTT alterations at any point during pre-filter UFH therapy. These non-mutually exclusive alterations included 16 patients with an aPTT greater than two times baseline, 22 patients with a therapeutic aPTT, and seven patients with a supratherapeutic aPTT. Baseline characteristics can be found in Table 1.

**Figure 1:**
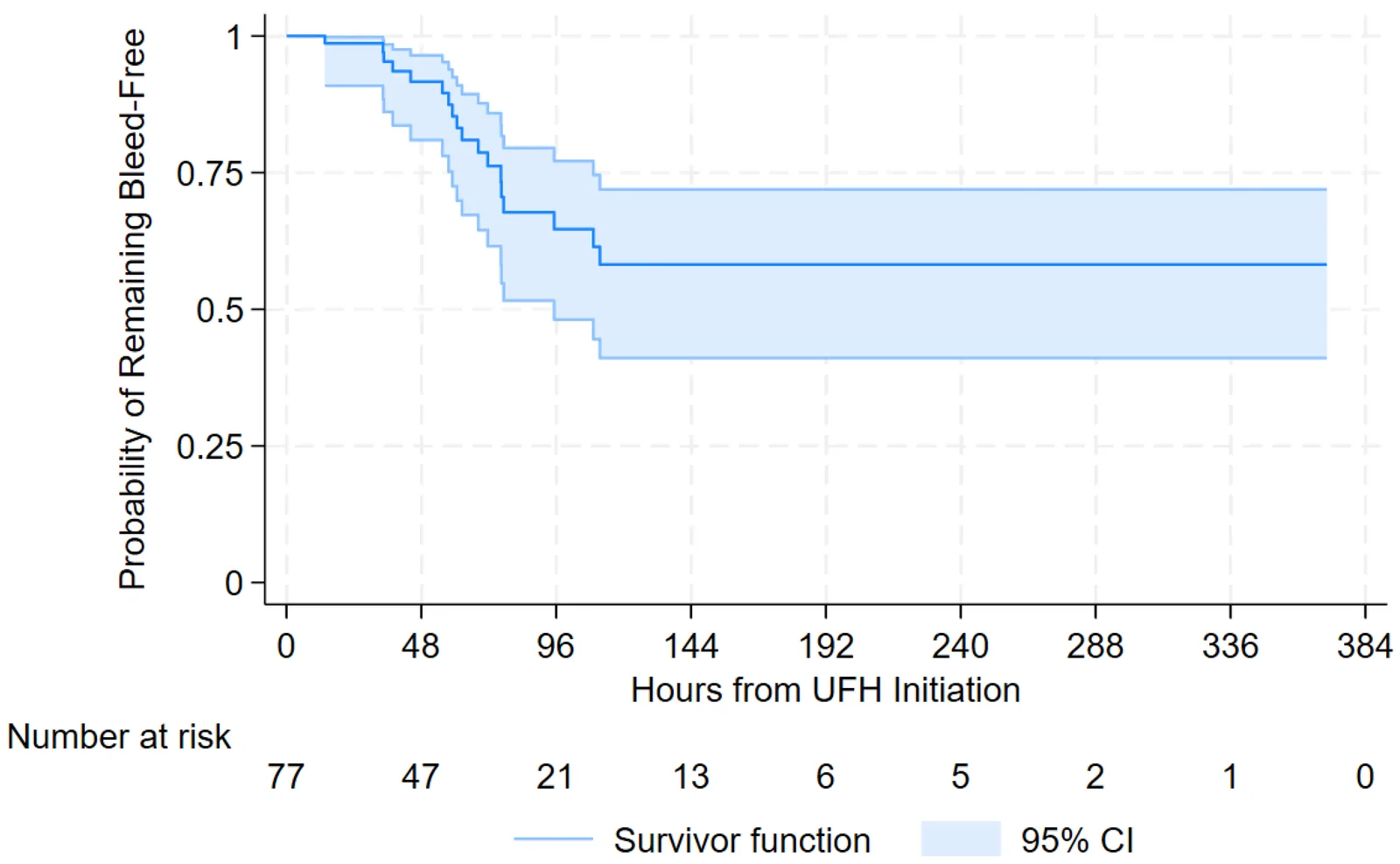
Kaplan–Meier curve of time to first clinically relevant major bleeding event. UFH = unfractionated heparin.

Clinically relevant major bleeding events occurred in 18 patients (23%), with a median time to first bleeding event of 61.5 h (IQR 44–76) among those affected. In the time-to-event analysis of the full cohort, the cumulative probabilities of bleeding at 24, 48, 72, and 96 h were 1.3%, 8.4%, 23.8%, and 35.3%, respectively. The Kaplan–Meier median time to bleeding was not estimable in this analysis because less than half of the cohort experienced a bleeding event. Three patients experienced a second bleeding event. At the first bleeding event, the median weight-adjusted pre-filter UFH dose was 542 units/h with a median aPTT of 38 s. Bleeding event characteristics are detailed in Table 2 and Figure 1.

In exploratory unadjusted analyses, no patient or treatment characteristics were significantly associated with clinically relevant major bleeding (Table 3). Given the time-dependent nature of UFH exposure, a post hoc 48 h landmark sensitivity analysis was also performed. Among 47 patients on pre-filter UFH and without bleeding events at 48 h, 13 (28%) subsequently experienced a bleeding event.

**Figure 2:**
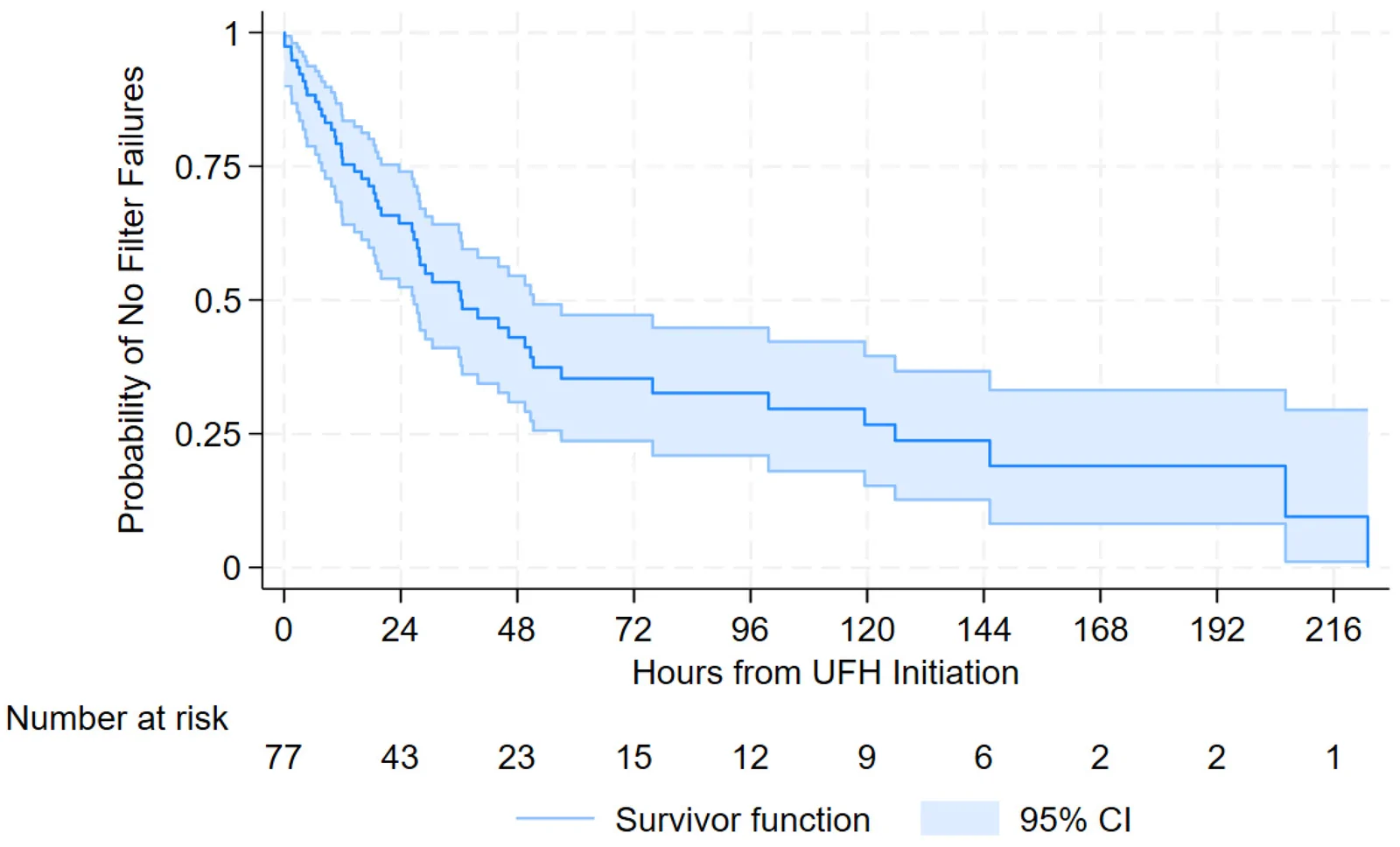
Kaplan–Meier curve of time to first CRRT filter failure. UFH = unfractionated heparin.

Fifty-one patients (66%) had at least one CRRT filter failure, with an average of 2.3 failures per patient among this group. In the time-to-event analysis of the full cohort, the median time to first filter failure was 36 h (95% CI: 27–51) (Figure 2). At the first filter failure, the median weight-adjusted pre-filter UFH dose was 516 units/h (IQR 411–710) with a median aPTT of 38 s (IQR 31–41). In a post hoc sensitivity analysis restricted to confirmed filter clotting, 14 patients (18%) experienced a documented clot. The estimated cumulative probabilities of documented filter clotting at 24, 48, 72, and 96 h were 11.6%, 18.9%, 18.9%, and 18.9%, respectively. Filter failure characteristics are detailed in Table 4.

## 4. Discussion

In this retrospective analysis evaluating the safety of pre-filter UFH for CRRT clotting prevention in critically ill patients, clinically relevant major bleeding occurred in 18 patients (23%). In the corrected time-to-event analysis, the cumulative probability of bleeding increased over time following pre-filter UFH initiation. Among the patients who remained on pre-filter UFH at 48 h without a bleeding event, 13 of 47 (28%) subsequently experienced bleeding. Furthermore, in unadjusted exploratory analyses of patient- and treatment-related characteristics, no covariates were significantly associated with bleeding. While these findings describe the frequency and timing of bleeding during pre-filter UFH therapy at our institution, they do not establish any causal relationship for treatment duration, dose, or other characteristics that were collected.

Unlike studies focused on protocolized anticoagulation strategies or alternative modalities such as RCA, the present study reflects real-world pre-filter UFH use in a heterogeneous ICU population selected at the discretion of treating clinicians and nephrologists for perceived risk of or recurrent circuit clotting. In addition, the present study differs from previous studies in that bleeding events were manually identified through chart review and using modified ISTH definitions rather than comparing transfusion requirements. Our institutional practice typically uses a fixed weight-based pre-filter UFH infusion, whereas other institutions may employ titrations based on target aPTT ranges. Rognoni et al. performed a meta-analysis of prospective and retrospective studies comparing pre-filter UFH with RCA for CRRT filter clotting and reported a lower incidence of bleeding events (12.6%), shorter average filter lifespan (16.43 h, compared to a median in our study), and less filter clotting (50.7%, compared to the filter failure classification in our study) with the use of RCA [17]. Phung et al. performed a retrospective analysis characterizing pre-filter UFH trends at their institution and demonstrated a shorter median duration of pre-filter UFH (52.4 h) but a similar median weight-adjusted pre-filter UFH dose (500 units/h) compared to the present study [18]. Lastly, in the pre-filter UFH arm of the recent ORCA trial, the incidence of overt bleeding was higher (40.3%) and the median filter lifespan was longer (36 h), although bleeding definitions, patient populations, CRRT protocols, and anticoagulation strategies differed from those in the present study [13].

The temporal distribution of bleeding warrants consideration. In the original exploratory analysis, multiple pre-filter UFH duration thresholds were associated with bleeding. However, the total pre-filter UFH duration accumulates after treatment initiation and may inherently be influenced by bleeding, treatment discontinuation, clinical deterioration, or CRRT duration. Therefore, classifying patients based on their eventual pre-filter UFH duration introduces temporal bias and may not establish a true duration threshold in which pre-filter UFH increases bleeding risk. In the revised analysis, patients without bleeding were censored at pre-filter UFH discontinuation, and the cumulative bleeding probability increased over time following UFH initiation. Consistently with this observation, in the post hoc 48 h landmark analysis, bleeding continued to occur after 48 h in patients who remained on therapy, though this was analyzed descriptively without a comparison group so a causal effect cannot be inferred. Overall, these findings support close monitoring for bleeding events in patients who require prolonged pre-filter UFH exposure, rather than determining a specific duration cutoff.

aPTT-related variables were not significantly associated with bleeding in exploratory analyses, though this should be interpreted cautiously. aPTT measurements were generally obtained daily as part of routine clinical care rather than at standardized intervals, and additional measurements could be obtained depending on the clinical context. Furthermore, aPTT abnormalities occurred at any point during pre-filter UFH therapy, which does not account for the timing or duration of the abnormality in relation to bleeding. Patients receiving pre-filter UFH for longer durations may have had more opportunities to have an abnormal aPTT documented. These aPTT-related limitations thus prevent conclusions regarding the utility of aPTT in predicting bleeding events during pre-filter UFH therapy.

CRRT filter failure was common, occurring in 51 patients (66%), with a corrected median time to first filter failure of 36.4 h. However, 37 of the 51 first recorded filter failures were characterized as unsuccessful rinsebacks, and the remaining 14 were confirmed filter clots. In a post hoc time-to-event sensitivity analysis of the 14 confirmed circuit clots, the estimated cumulative probabilities of clotting were 11.6% at 24 h and 18.9% at 48 h. The difference in frequency between unsuccessful rinsebacks and confirmed filter clotting suggests that unsuccessful rinsebacks may not be specific for circuit clotting and may thus overestimate the frequency of true filter clotting. Regardless, unsuccessful rinsebacks are clinically relevant in this context because they may disrupt CRRT even when circuit clotting is not directly documented.

Several additional limitations merit consideration. As with any retrospective study, causality cannot be established, and there is the potential for residual confounding. Since pre-filter UFH initiation was determined based on the nephrologist and primary team’s clinical judgement and reserved for specific critically ill patients, there was a significant risk of selection bias, and this study cohort may not represent all patients receiving CRRT. Additionally, the absence of a comparator arm prevents conclusions regarding the absolute safety of pre-filter UFH. Clinically relevant major bleeding occurred in approximately one-quarter of the cohort, so the present study should be interpreted as describing an observed bleeding frequency rather than establishing true safety. The small sample size and number of bleeding events reduced the statistical power. The exploratory analyses were unadjusted, and clinically relevant determinants of bleeding may have been undetected as a result. Albumin was not collected, and liver dysfunction, a risk factor for bleeding, was captured only by a pre-defined AST or ALT threshold rather than other assessments. Other confounding factors that were unaccounted for include individual variation in filter characteristics, blood flow rates, and other CRRT-specific factors that may have altered circuit patency. Furthermore, this study did not assess thrombotic outcomes.

The modified ISTH definition identified bleeding events temporally associated with pre-filter UFH but could not establish that UFH itself contributed to the event. While the exclusion of events occurring within 6 h of an operating-room procedure may have reduced the likelihood of capturing a procedural-related bleed, there were still several events documented as procedural or wound-related bleeds; the modified criteria do not establish that UFH contributed to the causality of these events. Six bleeding events had no documented bleeding site but met the objective criteria for a bleed based on hemoglobin decrease and/or transfusion requirements. Bleeding event adjudication was performed based on the consensus of three pharmacists, but formal inter-rater agreement was not assessed. All of these factors should be considered when interpreting the 23% bleeding incidence in this study.

Hospital mortality was high at 46%, which likely reflects the severity of illness in this patient population and may limit its generalizability to all CRRT recipients. In the revised time-to-event analyses, follow-up was censored at the end of each patient’s eligible treatment period rather than at death, as no patient’s eligible treatment period ended because of death. Additionally, the time of death was not available; therefore, a formal competing-risk analysis for death was not performed. In this context, censoring at pre-filter UFH discontinuation may also have been informative if clinical deterioration, concern for bleeding, or changes in CRRT influenced treatment discontinuation.

Finally, KDIGO guidance favors RCA for CRRT anticoagulation when it is feasible and not contraindicated [3]. The present study’s cohort does not provide evidence to support or refute this guidance, especially given the small sample size and observed frequencies of bleeding and filter failure. Instead, this study provides real-world characterization of pre-filter UFH use and may help inform monitoring and reassessment for clinicians who have opted to use pre-filter UFH.

Despite these limitations, this study provides clinically relevant insights into pre-filter UFH dosing, bleeding timing, aPTT patterns, and CRRT circuit outcomes in critically ill patients receiving pre-filter UFH for CRRT circuit clotting prevention. Future studies should aim to evaluate pre-filter UFH in larger, multicenter cohorts using standardized bleeding and circuit clotting definitions in a prospective manner to explore whether alternative pre-filter UFH administration, dosing, or monitoring strategies may mitigate bleeding risk while maintaining efficacy when RCA is not an option.

## 5. Conclusions

In this retrospective study of critically ill patients receiving pre-filter UFH for CRRT clotting prevention, clinically relevant major bleeding occurred in 23% of patients and continued to occur with longer durations of pre-filter UFH exposure. There were no patient- or treatment-related characteristics associated with bleeding in any exploratory analyses, including alterations in aPTT. Continuous renal replacement therapy filter failure was common, although the majority of first filter failures were unsuccessful rinsebacks rather than confirmed filter clots. Pre-filter UFH dose adjustments were infrequent, typically involving dose reductions, and often following filter failure. These findings provide a real-world characterization of pre-filter UFH use and associated bleeding and CRRT circuit outcomes but do not establish the comparative safety of this anticoagulation strategy. Larger, prospective studies are warranted to further characterize bleeding risk and to refine pre-filter UFH dosing and monitoring approaches to balance safety and efficacy in this population.

## Figures and Tables

**Table 1:** Baseline characteristics and overall trends.

Characteristic	Pre-Filter UFH (*n* = 77)
Age, years, median (IQR)	61 (47–70)
Sex, *n* (%)	
Female	26 (34)
Male	51 (66)
Race, *n* (%)	
White	48 (62)
Black	24 (31)
Other	5 (7)
Weight, kilograms, median (IQR)	91 (77–120)
Intensive Care Unit, *n* (%)	
Cardiovascular surgical	27 (35)
Medical	24 (31)
Surgical	12 (16)
Cardiac	7 (9)
Burn	3 (4)
Neurocritical care	2 (3)
Oncology	2 (3)
Potential Bleeding-Related Risk Factors, *n* (%)	
VTE prophylaxis	33 (43)
Antiplatelet therapy	20 (26)
Platelets < 50 × 10^3^/mm^3^	27 (35)
AST or ALT > five times ULN	6 (8)
Hospital Mortality	35 (46)
Pre-filter UFH Starting Dose, *n* (%)	
6 units/kg/h	69 (90)
4 units/kg/h	3 (4)
5 units/kg/h	3 (4)
4.5 units/kg/h	1 (1)
7 units/kg/h	1 (1)
Weight-Adjusted Pre-filter UFH Starting Dose, units/h, median (IQR)	535 (462–706)
Pre-filter UFH Duration, h, median (IQR)	72 (37–137)
Pre-filter UFH Dose Adjustment, *n* (%)	20 (26)
Increase	7 (35)
Decrease	13 (65)
aPTT Alterations, *n* (%) *	
Any alteration	24 (31)
Any aPTT > two times baseline	16 (21)
Any therapeutic aPTT (50–80.9 s)	22 (29)
Any supratherapeutic aPTT (>80.9 s)	7 (9)

UFH = unfractionated heparin; IQR = Interquartile Range; VTE = venous thromboembolism; AST = aspartate aminotransferase; ALT = alanine aminotransferase; ULN = upper limit of normal; aPTT = activated partial thromboplastin time. * aPTT alterations were not mutually exclusive, and patients could meet more than one aPTT criterion during therapy.

**Table 2:** Bleeding events.

Characteristic	Bleeding Events (*n* = 18)
First Bleeding Event Criterion Met, *n* (%)	
Transfusion of ≥2 units RBCs	11 (61)
Fall in hemoglobin ≥2 g/dL & transfusion of ≥2 units RBCs	4 (22)
Critical area or organ [retroperitoneal]	2 (11)
Fall in hemoglobin ≥2 g/dL	1 (6)
First Bleeding Event Location, *n* (%)	
No documented bleeding site *	6 (33)
Gastrointestinal	3 (17)
Retroperitoneal/Abdominal wall	3 (17)
Procedural/Wound-related	3 (17)
Upper airway/ENT	2 (11)
Gynecological	1 (5)
Timing of Pre-filter UFH Dose Adjustment in Patients with Both a Bleeding Event and a Dose Adjustment, *n* (%) **	
Before first bleed	5 (83.3)
After first bleed	1 (16.7)
Pre-Filter UFH Dose at First Bleeding Event, units/kg/h, median (IQR)	6 (6–6)
Weight-Adjusted Pre-Filter UFH Dose at First Bleeding Event, units/h, median (IQR)	542 (308–708)
aPTT Nearest the First Bleeding Event, seconds, median (IQR)	38 (31–41)
Therapeutic or Supratherapeutic aPTT Nearest the First Bleeding Event, *n* (%)	3 (17)
Time to First Bleeding Event Among Patients with Bleeding, h, median (IQR)	61.5 (44–76)

RBCs = red blood cells; ENT = Ears, Nose, and Throat; UFH = unfractionated heparin; IQR = Interquartile Range; therapeutic aPTT defined as 50 to 80.9 s; supratherapeutic aPTT defined as >80.9 s. * Among the six bleeding events without a documented site, four met the red blood cell transfusion criterion only, one met the hemoglobin decrease criterion only, and one met both criteria. ** Patients with both a bleed and a pre-filter UFH dose change only included 6 patients.

**Table 3:** Exploratory unadjusted associations with clinically relevant major bleeding.

Variable, *n* (%) *	No Bleeding (*n* = 59)	Bleeding (*n* = 18)	Fisher’s Exact *p*-Value
Age ≥ 65 Years	22 (73)	8 (27)	0.59
Cardiac ICU **	26 (76)	8 (24)	1.00
Surgical ICU **	31 (74)	11 (26)	0.60
Any UFH Dose Adjustment	15 (71)	6 (29)	0.55
VTE Prophylaxis	25 (76)	8 (24)	1.00
Antiplatelet Use	14 (70)	6 (30)	0.54
AST or ALT > five times ULN	5 (83)	1 (17)	1.00
Platelet Count < 50 × 10^3^/mm^3^	19 (70)	8 (30)	0.40
Any aPTT Abnormality	16 (67)	8 (33)	0.24
Any aPTT > two times Baseline	11 (69)	5 (31)	0.51
Any Therapeutic aPTT	14 (64)	8 (36)	0.13
Any Supratherapeutic aPTT	6 (86)	1 (14)	1.00

ICU = intensive care unit; UFH = unfractionated heparin; VTE = venous thromboembolism; AST = aspartate aminotransferase; ALT = alanine aminotransferase; ULN = upper limit of normal; aPTT = activated partial thromboplastin time. * Percentages represent row percentages for each characteristic. ** ICU classifications were not mutually exclusive and were evaluated separately.

**Table 4:** CRRT filter failure characteristics.

Characteristic	Any CRRT Filter Failure (*n* = 51)
First Filter Failure Classification, *n* (%)	
Documented clot	14 (27)
Rinseback unsuccessful	37 (73)
Timing of Pre-filter UFH Dose Adjustment in Patients with Both a Filter Failure and a Dose Adjustment, *n* (%) *	
Before first filter failure	7 (41)
After or same time as first filter failure	10 (59)
Filter Failures Per Patient Among Patients with a Filter Failure, mean (SD)	2.3 (1)
Weight-Adjusted UFH Dose at First Filter Failure, units/h, median (IQR)	516 (411–710)
aPTT at First Filter Failure, seconds, median (IQR)	38 (31–41)
Time to First Filter Failure, h, median (95% CI)	36 (27–51)

SD = Standard Deviation; UFH = unfractionated heparin; IQR = Interquartile Range; aPTT = activated partial thromboplastin time; CI = Confidence Interval. * Patients with both a filter clot and a pre-filter UFH dose change only included 17 patients.

## Data Availability

The raw data supporting the conclusions of this article will be made available by the authors on request.
